# Development of energy plants from hybrids between *Miscanthus sacchariflorus* and *M. lutarioriparius* grown on reclaimed mine land in the Loess Plateau of China

**DOI:** 10.3389/fpls.2022.1017712

**Published:** 2023-01-16

**Authors:** Xuhong Zhao, Liang Xiao, Jia Mi, Lifang Kang, Cong Lin, Wenli Chen, Hongmei Huang, Juan Yan, Zili Yi, Tao Sang, Wei Liu

**Affiliations:** ^1^Key Laboratory of Plant Resources and Beijing Botanical Garden, Institute of Botany, Chinese Academy of Sciences, Beijing, China; ^2^College of Life Sciences, University of Chinese Academy of Sciences, Beijing, China; ^3^College of Bioscience and Biotechnology, Hunan Agricultural University, Changsha, Hunan, China; ^4^Shanxi Key Laboratory for Ecological Restoration of Loess Plateau, Institute of Loess Plateau, Shanxi University, Taiyuan, Shanxi, China; ^5^State Key Laboratory of Systematic and Evolutionary Botany, Institute of Botany, Chinese Academy of Sciences, Beijing, China; ^6^Key Laboratory of Plant Germplasm Enhancement and Specialty Agriculture, Wuhan Botanical Garden, Chinese Academy of Sciences, Wuhan, Hubei, China; ^7^Key Laboratory of Dryland Agriculture, MOA, Institute of Environment and Sustainable Development in Agriculture, Chinese Academy of Agricultural Sciences, Beijing, China

**Keywords:** *Miscanthus sacchariflorus*, *Miscanthus lutarioriparius*, hybrids, reclaimed mine land, biomass, photosynthetic rate, water use efficiency

## Abstract

*Miscanthus*, a promising bioenergy plant, has a high biomass yield with high cellulose content suitable for biofuel production. However, harsh climatic and poor soil conditions, such as barren lands or abandoned mines, pose a challenge to the survival and yield of *Miscanthus* feedstock on the marginal land. The selection from the interspecific hybrids of *Miscanthus* might combine high survival rates and high yield, which benefits energy crop development in multi-stressful environments. A total of 113 F_1_ hybrids between *Miscanthus sacchariflorus* and *M. lutarioriparius* together with the parents were planted and evaluated for multiple morphological and physiological traits on the mine land of the Loess Plateau of China. The majority of hybrids had higher establishment rates than *M. sacchariflorus* while *M. lutarioriparius* failed to survive for the first winter. Nearly all hybrid genotypes outperformed *M. lutarioriparius* for yield-related traits including plant height, tiller number, tiller diameter, and leaf area. The average biomass of the hybrids was 20 times higher than that of surviving parent, *M. sacchariflorus*. Furthermore, the photosynthetic rates and water use efficiency of the hybrids were both significantly higher than those of the parents, which might be partly responsible for their higher yield. A total of 29 hybrids with outstanding traits related to yield and stress tolerance were identified as candidates. The study investigated for the first time the hybrids between local individuals of *M. sacchariflorus* and high-biomass *M. lutarioriparius*, suggesting that this could be an effective approach for high-yield energy crop development on vast of marginal lands.

## Introduction

*Miscanthus* is a promising energy crop that has high biomass and high cellulose content suitable for the production of biofuels (Xiang et al., 2020). As a C4 perennial grass with high nutrient and water use efficiencies, *Miscanthus* can be grown on the marginal land in the cool climate without heavy irrigation or fertilization (Yan et al., 2012). In addition, growing *Miscanthus* has a lot of environmental benefits, including soil and water conservation, carbon sequestration and soil restoration in the marginal land (Liu et al., 2014; Mi et al., 2014; Jezowski et al., 2017; Mi et al., 2018). Furthermore, the strong resistance to low temperature has also been reported in *Miscanthus* species (Fonteyne et al., 2016). These traits give *Miscanthus* an obvious advantage to ensure the high survival rate and biomass yields in harsh habitats. However, whether *Miscanthus* can adapt to multi-stressful environments and provide sustainable feedstocks for lignocellulosic biofuels remains unclear. This kind of typical marginal land under multiple stresses is infertile reclaimed mine lands with low temperature and drought.

In recent years, the mining was carried out around the world, which caused a large amount of destroyed natural vegetation and finally turned into the abandoned wasteland (Festin et al., 2019). It was reported that there was 3.2 Mha of wasteland caused by mining at the end of 2004 in China and that was increasing at a rate of 46,700 ha per year (Li, 2006). However, the overall restoration rate of abandoned mine lands was between 10%~12%, although the restoration of abandoned mine lands was first implemented in late 1970 (Li, 2006). There were still a lot of wastelands representing vast areas of marginal lands (Li, 2006; Li et al., 2007), especially in those open-cast mining areas in northern China. The largest open-cast coal mine in Asia is located on the Loess Plateau of China. The severe conditions in these areas, such as the cold winter and little rainfall, could pose a major challenge to the survival and growth of *Miscanthus* (Yan et al., 2012).

Low temperature was one of the important limiting factors for *Miscanthus* survival and growth (Dong et al., 2019). Most *Miscanthus* genotypes can recover from a mild low-temperature above zero (Fonteyne et al., 2016). The minimum temperature that 50% *M. × giganteus* and *M. sacchariflorus* genotypes can tolerate was around -3.4°C (Clifton-Brown and Lewandowski, 2000), while hybrids can survive temperatures as low as -14°C (Fonteyne et al., 2016). During the first winter following establishment cold winters can kill young plants that have underdeveloped rhizomes and the biomass of *Miscanthus* was often lower than its potential yield (Clifton-Brown et al., 2001). *M. × giganteus*, a natural hybrid by *M. sacchariflorus* and *M. sinensis*, is widely recognized as a promising energy crop for biofuel production (Zeng et al., 2020) but insufficient winter rhizome cold tolerance limited its production in a colder habitat (Clifton-Brown and Lewandowski, 2002).

Similarly, drought has hampered the survival and growth of *Miscanthus* (Van der Weijde et al., 2017; De Vega et al., 2021). The decreases in stem elongation rate and photosynthetic performance were observed successively when *M. × giganteus* was suffering from drought stress (Ings et al., 2013). It could lead to a decrease in stomatal conductance, chlorophyll fluorescence and chlorophyll content of the leaf, which caused a further reduction in photosynthetic rate and consequently carbon starvation (Ings et al., 2013). A recent study indicated that drought stress could reduce plant weight by 45% and cause a large variation between *Miscanthus* genotypes (Van der Weijde et al., 2017).

Faced with these adverse environmental conditions, it was expected to select more *Miscanthus* species to breed interspecific hybrid with stronger adaptability and higher biomass (Stavridou et al., 2019). A study found that cold-tolerant *Miscanthus* genotypes exhibited richer key photosynthetic enzyme and thus higher photosynthetic activity under low-temperature stress (Friesen and Sage, 2016). In addition, it has been reported that some *M. sacchariflorus* genotypes had strong tolerance to cold temperature (Yan et al., 2012), which may be useful for breeding new *Miscanthus* crops with improved cold and drought tolerance. Therefore, *Miscanthus* hybrids, especially interspecific hybrids, were expected to have more outstanding growth traits and higher survival rate than their parents in the stressful environment.

In order to make a use of marginal land on the Loess Plateau of China, hybrids between *M. sacchariflorus* and *M. lutarioriparius* were obtained and grown on the mine land of the Loess Plateau. The female parent of *M. sacchariflorus* was from a population native to the Loess Plateau, adapted to the cold and drought environment but producing relatively low biomass (Yan et al., 2012). *M. lutarioriparius*, an endemic species in Central China with the highest biomass production, has been shown to have high water use efficiency and high yield in certain locations of the Loess Plateau where it was able to overwinter (Yan et al., 2015; Zhao et al., 2021).

A total of 113 F_1_ hybrids together with the parents were planted at an infertile mine site on the Loess Plateau of China. Because the *Miscanthus* species are self-incompatible (Yan et al., 2016), such high heterozygosity ensured that each F_1_ hybrid was genetically unique and consequently a sufficient number of genotypes were evaluated. Multiple clonal rhizomes from each genotype were grown to allow the evaluation of establishment rates. For two consecutive growth seasons, morphological and physiological traits related to yield and stress tolerance were measured and analyzed. A number of hybrid genotypes that not only outperformed their parents but exhibited outstanding traits were identified and formed the basis for future energy crop development in the vast area of marginal lands with harsh climates and soil conditions.

## Materials and methods

### The basic situations of the experimental site

The field experiment was established on the flat land covered by reclaimed soil in the mine of aluminium ore mine at Xiaoyi (37.12°N, 111.45°E) in Shanxi Province, which is located on the Loess Plateau of China. The reclaimed soil was identified to have poor nutrients at the approximately 0-40 cm depth. The original average soil organic carbon and total nitrogen were 3.61 g/kg and 0.33 g/kg before planting *Miscanthus*, respectively. Annual and perennial weeds such as Artemisia and reed grass, and so on covered the area. Weed control was made only at the beginning of the first growing season in July 2018. Fertilizer was not applied in the experimental field during the whole planting and growing period of *Miscanthus*. The average annual precipitation was 200~750 mm, the average annual temperature was -10~15°C in the past 50 years in the Loess Plateau of China (Sun et al., 2015). Daily weather data including temperature and precipitation from November 2017 to November 2019 in the experimental area were obtained from the meteorological station near the experimental field run by the China Meteorological Data Sharing Service System (http://data.cma.cn). Annual precipitation was 388 mm in 2018, of which 172 mm was concentrated in August. Meanwhile, annual precipitation was 443 mm in 2019, of which 209 mm was concentrated in August and September. The daily minimum ground temperature was below 0°C for 148 days from October 2018 to April 2019 and was below -15°C for 19 days from December 2018 to February 2019, respectively. The daily maximum air temperature was below 10°C for 105 days from November 2018 to March 2019.

### Experimental design

The female parent *M. sacchariflorus* with cold tolerance but relatively low biomass was selected from Yaodian in Gansu Province (35.19°N, 107.23°E) in the Loess Plateau. The male parent *M. lutarioriparius* for breeding was selected from Changsha in Hunan Province (28.12°N, 112.59°E). Previous studies indicated that *M. lutarioriparius*, an endemic species in Central China, has a higher water use efficiency and the highest biomass production among *Miscanthus* species (Yan et al., 2012; Fan et al., 2015). Also, growing this high-biomass plant in the Loess Plateau was found to have positive environmental impacts, such as soil and water conservation, carbon sequestration, and soil restoration in the infertile and soil-eroded region (Liu et al., 2016). The hybrid experiment was conducted in October 2010 at Hunan Agricultural University (Ai et al., 2017).

A total of 113 F_1_ hybrid genotypes selected at random and together with their parents were propagated by clonal rhizomes to Xiaoyi in May 2018. The same quantity of 7~10 cm of 10 clonal rhizomes for each hybrid genotype and 15 clonal rhizomes for each parent were planted in the homogeneously reclaimed soil with a depth of 5 cm and a row spacing of 1 m × 1 m. The experiment used a completely random design. 1160 individuals with a planting density of 1 m^2^ per individual were planted 1 m apart in a 60 × 20 m layout according to the order of individuals in the randomized table. The rhizomes were watered before soil filled the planting hole and they were not irrigated again after that.

### Phenotype traits measurements of *Miscanthus*

For two consecutive growth seasons from 2018 to 2019, morphological and physiological traits related to yield and stress tolerance were measured and analyzed. *Miscanthus* that grew at the end of the 2019 season were all robust and thus considered to be established from the point of view of energy crop production. The adaptability of *Miscanthus* was assessed by the establishment rate, survival rate, and overwinter rate. The survival rate was the ratio of surviving rhizomes at the end of the 2018 growing season to the number of rhizomes planted at the beginning of the 2018 growing season. Overwinter rate was the ratio of surviving rhizomes in June 2019 growing season to the number of surviving rhizomes at the end of the 2018 growing season. The establishment rate was the ratio of surviving rhizomes at the end of the 2019 growing season to rhizomes planted at the beginning of 2018 growing season (Yan et al., 2012).

Yield-related traits included plant biomass (the weight of all tillers within 1 m^2^ for each plant), individual tiller’s biomass (the weight of a single tiller), plant height (the length from the soil surface to the youngest leaf with a ligule on the tallest stem for each plant) and stem height (the length from the soil surface to the point on the stem that was subtended by the ligule of the youngest differentiated leaf). Stem traits included node number (the count of the nodes in the stem) and tiller number (stem number within 1 m^2^ around the plant), stem diameter (the diameter of the stem at 3-5 cm from the ground), internode length (the length of the largest internode in the stem), and branch number (the number of branches that set in the node). Leaf traits included leaf area (the area of flat blade from the auricle to the top), leaf width (width of the leaf blade at approximately half leaf length), and the leaf base angle (the angle between the base of leaf vein and the stem). The biomass and internode length were measured in November 2018 and 2019. Leaf area was measured using leaf photographs once a month from July to September in 2018 and 2019 by the Digimizer image analysis software (MedCalc Software Ltd, Belgium). The leaf base angle of the fourth leaf from the plant top was measured once a month from August to October in 2019 by the protractor tool. Other traits were measured once a month from June to October for two years. All of these traits were measured based on three individuals sampled randomly for each genotype. The three individuals were used subsequently measurement throughout two growing seasons.

### Photosynthetic parameters measurement and water use efficiency analysis

The photosynthetic parameters, including CO_2_ fixation rate (*A*), stomatal conductance (*g*_s_), intercellular CO_2_ concentration (*C_i_*), and transpiration rate (*E*), were measured in the field using LI-6400 portable photosynthesis system (LI-COR 6400 XT system; LI-COR, Lincoln, NE, USA) connected to a standard 6 cm^2^ cuvette. Instrument warm-up was conducted for 20 min while routine examination and calibration were done. The middle part of the fourth leaf from top to bottom in the canopy was enclosed in the leaf chamber and logged after readings reached stability (1-2 min) (Fan et al., 2015). The infrared gas analyzer (IRGA) was matched to reach equilibrium (Monitor ΔCO_2_ and ΔH_2_O) at a 20-min interval. Each match took 2-3 minutes. Measurements were allowed to track the ambient temperature and photon flux density, while the CO_2_ mixer was used to maintain the reference CO_2_ concentration at 400 µmol mol^-1^. The process was carried out between 10:00 hours and 12:00 hours, and each plant was logged three times for measurement (Yan et al., 2015). These measurements were taken on 1-6 Jul., 18-27 Aug., 22-28 Sep. 2018 and on 23-25 Jul., 17-18 Aug., 21-24 Sep. 2019.

The fluorescence parameters included the excitation energy capture efficiency (*Fv’/Fm’*) of the open PSII reaction center under light, the actual photochemical quantum efficiency (*ΦPSII*) of PSII in the presence of applied light, and the quantum yield (*Φ_CO2_*) corresponding to the rate of CO_2_ assimilation, photochemical quenching parameter (*qP*), non-photochemical quenching coefficient (*NPQ*), charge separation by absorption of light energy by the photosynthetic mechanism, and the rate (*ETR*) of electrons passing down the electron transfer chain (Yan et al., 2015). A total of 30 genotypes were randomly selected and marked from 113 hybrid genotypes for detecting the fluorescence parameters of leaves during the growing season. The fluorescence parameters were measured between 10:00 hour and 1:00 hour under the light-adapted condition with the leaf chamber fluorometer (6400-40, LI-COR) on 27 and 28 Aug. 2018, and 21 and 27 Aug. 2019.

Water use efficiency was calculated from the ratio of the photosynthetic rate to the transpiration rate (WUE_e_, *A/E*) and the ratio of the photosynthetic rate to stomatal conductance (WUE_i_, *A/g_s_*), respectively. The former (WUE_e_) represents the external water use efficiency, which reflects the CO_2_ amount fixed by plant leaf when 1 mol H_2_O was transpired (Beer et al., 2009). The latter (WUE_i_) represents the intrinsic water use efficiency and is related to the change of leaf stomatal conductance. It reflects the relationship between the degree of leaf stomatal conductance and the CO_2_ amount fixed (Beer et al., 2009).

### Data analysis

Student’s *t* test was used to compare survival rate, overwinter rate, and establishment rate of hybrids and the parents in the 2018 and 2019 growing seasons. Two-way ANOVA analysis was used to compare morphological and physiological traits of hybrid genotypes and the parents in different growth months and years, respectively. Multiple linear regression was used to identify the key growth traits related to the plant biomass and biomass of individual tiller. Correlation analysis was used to analyze the relationship between growth traits and photosynthetic parameters. All statistical analyses were conducted using R (version 3.6.2).

To identify the hybrids with outstanding traits related to yield and stress tolerance, one concise method was to compare the total biomass of all survival individuals for each hybrid in November 2019. To include more morphological traits, three other evaluation methods were used, including membership function analysis, principal component analysis, and cluster analysis. By comparing the selected hybrid genotypes from the four methods above, the intersect was used to identify as candidates that performed well on the mine land of the Loess Plateau.

In membership function analysis, the score of growth trait for each hybrid was calculated by the following formula (Chen et al., 2012):


$$R_{i}=\frac{\left(X_{ij}-X_{j\;min}\right)}{\left(X_{j\;max}-X_{j\;min}\right)}$$

where *R*_i_ was the score of growth trait *j* for hybrid genotype *i*. *X*_ij_ was the value of growth trait *j* for hybrid genotype *i*. *X*_j max_ was the maximum value within the growth trait *j*. *X*_j min_ was the minimum value within the growth trait *j*.

The integrated score for each genotype was calculated by summing the score of all the growth traits. Sorting the integrated scores of different hybrid genotypes can obtain the genotypes with the best comprehensive traits.

When the integrated score of growth traits for each hybrid was calculated using principal component analysis (Ouyang, 2005), the following formula was


$$Y_{m}=\;\sum_{n=1}^{3}W_{n}F_{n}$$

where *Y*_m_ was the integrated score of the first three principal components for genotype *m*. *F*_n_ was the score of principal component *n*n. *W*_n_ was the weight of the principal component *n*.

The candidate hybrid genotypes can be gained by the rank of integrated score in the membership function and principal component analysis. Similarly, the systematical cluster analysis was used to identify the candidate hybrid genotypes and divided F_1_ hybrid genotypes into two categories, namely *M*_1_ and *M*_2_ with excellent and poor performance, respectively. One-way ANOVA was performed on the photosynthetic parameters between *M*_1_ and *M*_2_ genotypes to identify which photosynthetic parameters may contribute to the difference in growth performance.

## Results

### The adaptability of hybrids to stressful environments

Although the survival rate was 66.67% in the 2018 growing season, the male parent *M. lutarioriparius* failed to survive for the first winter so that the establishment rate was zero in the stressful site. For female parent *M. sacchariflorus*, survival rate, overwinter rate, and establishment rate were 33.33%, 60%, and 20%, respectively. For 113 F_1_ hybrids, the same indexes were 79.91%, 93.86%, and 65.75%, which were significantly higher than that of surviving parent *M. sacchariflorus* (*P*<0.01). Furthermore, there was a great between-genotype variation for hybrids. The survival rate showed the largest variation (29.3%) followed by establishment rate 11.28% while overwinter rate had the smallest variation (6.89%).

### Morphological traits of hybrids and the parents

**Figure 1:**
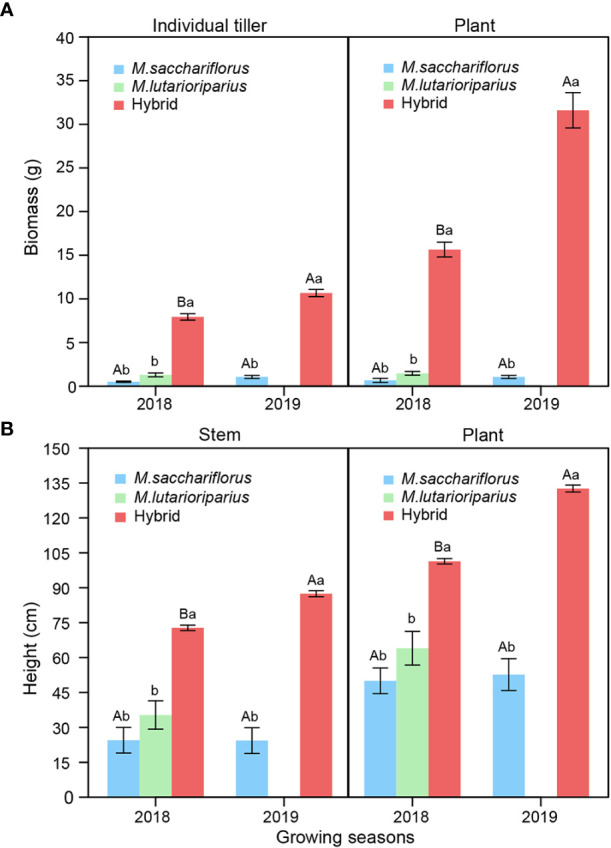
Differences of **(A)** average plant and individual tiller’ biomass, **(B)** average plant and stem height between measuring hybrid and parent individuals in two consecutive growth seasons. Different capital letters A and B represent a significant difference of plant and individual tiller’ biomass, and plant and stem height between two years at *P*<0.05 level, respectively. Different lowercases a and b represent a significant difference between *M. sacchariflorus*, *M. lutarioriparius* and their hybrids at *P*<0.05 level, respectively. Error bars indicate standard error of 333 surviving hybrid individuals, 5 and 3 surviving female parent individuals in both 2018 and 2019, and 10 surviving male parent individuals in 2018, respectively.

Both plant and individual tiller’s biomass of hybrids were significantly larger than those of *M. sacchariflorus* in both 2018 and 2019 growing season, and *M. lutarioriparius* in 2018 (*P*<0.05, **Figure 1A**). The average biomass of hybrids was 20 times higher than that of surviving parent, *M. sacchariflorus*. Similarly, the stem and plant height of hybrid genotypes were significantly higher than those of the female parent in both 2018 and 2019, and the male parent in 2018 (*P*<0.05, **Figure 1B**). In addition to these yield-related traits, hybrid genotypes outperformed the female parent in both 2018 and 2019, male parent in 2018 for other stem and leaf traits including tiller number, stem diameter, leaf area, and node number (**Supplementary Figure 1**). To reveal the establishing ability of perennial plants, these traits above, except for node number, in the second year were significantly better than those in the first year (*P*<0.05).

**Figure 2:**
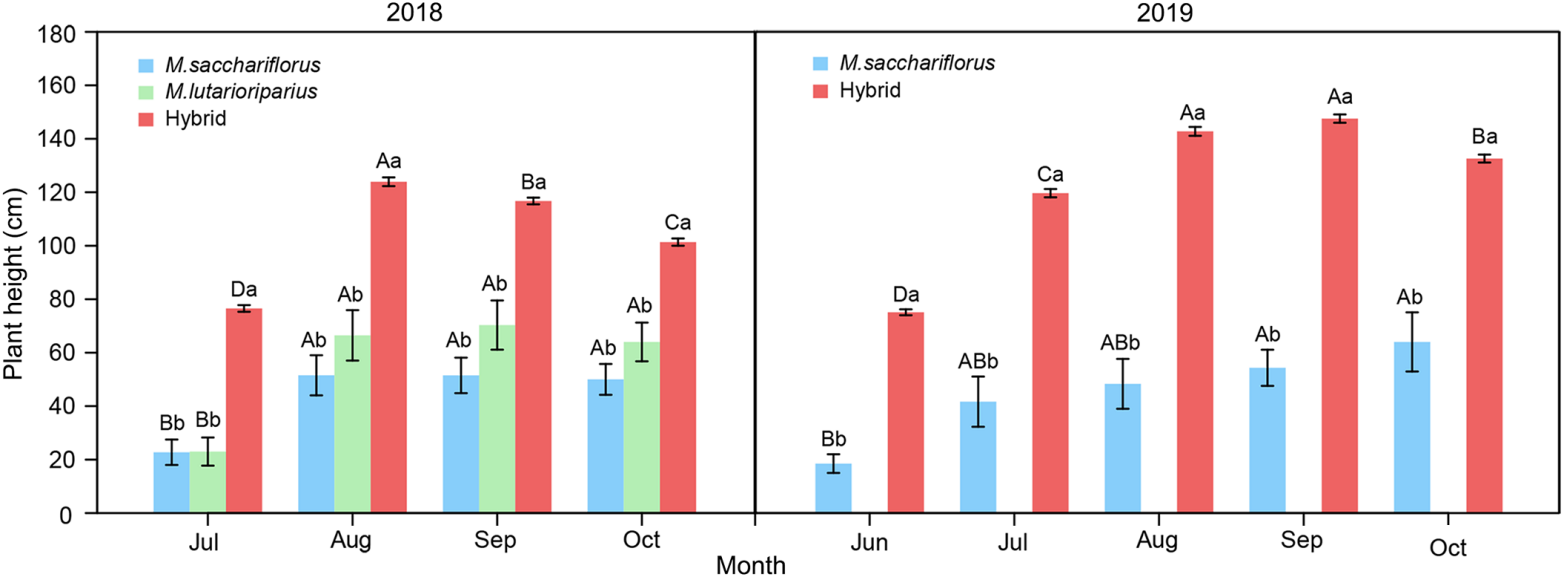
Differences of average plant height between measuring hybrid and female parent individuals throughout growth months in two growing seasons, and male parent individuals throughout growth months in the 2018 growing season. Different capital letters A, B, and C represent a significant difference of plant height between growth months at *P*<0.05 level, respectively. Different lowercases a and b represent a significant difference of *M. sacchariflorus*, *M. lutarioriparius* and their hybrids at *P*<0.05 level, respectively. Error bars indicate standard error of 333 surviving hybrid individuals, 5 and 3 surviving female parent individuals in both 2018 and 2019, and 10 surviving male parent individuals in 2018, respectively.

Within each growing season, the plant height of both hybrids and the female parent showed a trend of first increasing and then peaking in August or September. After that the plant height decreased in the following September (2018) or October (2019) (**Figure 2**). In contrast, the leaf width of hybrid genotypes showed different dynamic change in two growing seasons (**Supplementary Figure 2**). For each growing month of two years, the average plant height of hybrid genotypes was significantly higher than that of the female parent (*P*<0.05). A similar pattern in each growing month within each growing season was observed in tiller number, stem diameter, node number, and leaf width (**Supplementary Figures 2**, **3**).

### Physiological traits of hybrids and the parents

**Figure 3:**
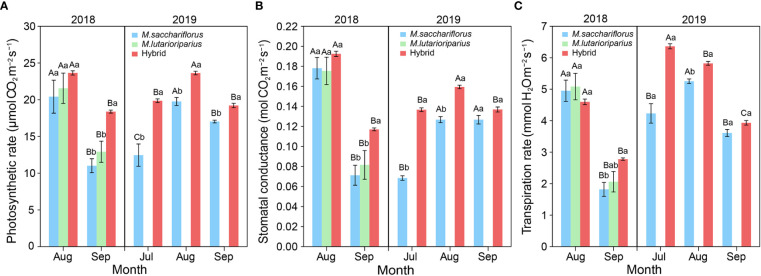
Differences of **(A)** average photosynthetic rate, **(B)** the advantage in average stomatal conductance, **(C)** transpiration rate between measuring hybrid and female parent individuals in both 2018 and 2019 growing season, and male parent individuals in 2018. Different capital letters A, B and C represent a significant difference of photosynthetic rate, stomatal conductance, and transpiration rate between growth months at *P*<0.05 level, respectively. Different lowercases a and b represent a significant difference of photosynthetic rate, and stomatal conductance, and transpiration rate between *M. sacchariflorus*, *M. lutarioriparius* and their hybrids at *P*<0.05 level, respectively. Error bars indicate standard error of 333 surviving hybrid individuals, 5 and 3 surviving female parent individuals in both 2018 and 2019, and 10 surviving male parent individuals in 2018, respectively.

The photosynthetic rate of hybrids and the female parent peaked in August and then decreased significantly in September in the 2018 and 2019 growing seasons (*P*<0.05, **Figure 3A**). Although there was no significant difference in photosynthetic rate between hybrids and the female parent in August in the first growing season, the photosynthetic rate of hybrids was significantly higher than that of the parents in cooler September 2018 (*P*<0.05). In 2019, the photosynthetic rate of hybrids was significantly higher than that of surviving parent *M. sacchariflorus* with each growing month (*P*<0.05, **Figure 3A**).

Similarly, the pattern above was observed in another key photosynthetic parameter, stomatal conductance. First, the peak in stomatal conductance of hybrids and the female parent occurred in August during the 2018 and 2019 growing seasons (**Figure 3B**). Then the stomatal conductance of hybrid genotypes significantly decreased from August to September in two years (*P*<0.05). Second, the stomatal conductance of hybrids was significantly higher than that of the parents in cooler September 2018 (*P*<0.05), but there was no significant difference between hybrids and the parents in August. Finally, the stomatal conductance of hybrids was significantly higher than that of surviving parent *M. sacchariflorus* in July and August of the 2019 growing season (*P*<0.05, **Figure 3B**). In contrast, the transpiration rate of hybrids significantly decreased in each growing season (*P*<0.05) while the transpiration rate of surviving parent *M. sacchariflorus* peaked in August 2019 (**Figure 3C**). And there was no significant difference in transpiration rate between hybrids and the parent genotypes in August 2018 and July, September 2019.

**Figure 4:**
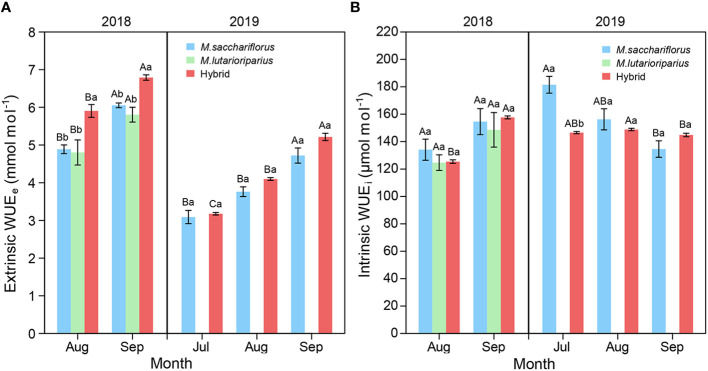
Differences of **(A)** average WUE_e_, **(B)** WUE_i_ between measuring hybrid and female parent individuals during the 2018 and 2019 growing season, and male parent individuals in 2018. Different capital letters A, B, and C represent a significant difference of WUE_e_ and WUE_i_ between growth months at *P*<0.05 level, respectively. Different lowercases a and b represent a significant difference between *M. sacchariflorus*, *M. lutarioriparius* and their hybrids at *P*<0.05 level, respectively. Error bars indicate standard error of 333 surviving hybrid individuals, 5 and 3 surviving female parent individuals in both 2018 and 2019, and 10 surviving male parent individuals in 2018, respectively.

Within each growing season, WUE_e_ of hybrids and the female parent significantly increased from July to September (*P*<0.05, **Figure 4A**). In 2018, WUE_e_ of hybrids was significantly higher than that of the parents in each growth month (*P*<0.05) while there was no significant difference between hybrids and the female parent in 2019. Likewise, WUE_i_ of hybrids and the parents showed an increasing trend in the 2018 growing season (**Figure 4B**). However, WUE_i_ of hybrids and the female parent significantly decreased in 2019. It was worth noting that WUE_i_ of surviving parent *M. sacchariflorus* was significantly higher than that of hybrids in July 2019 (*P*<0.05).

### Candidates for future production of biofuels

**Figure 5:**
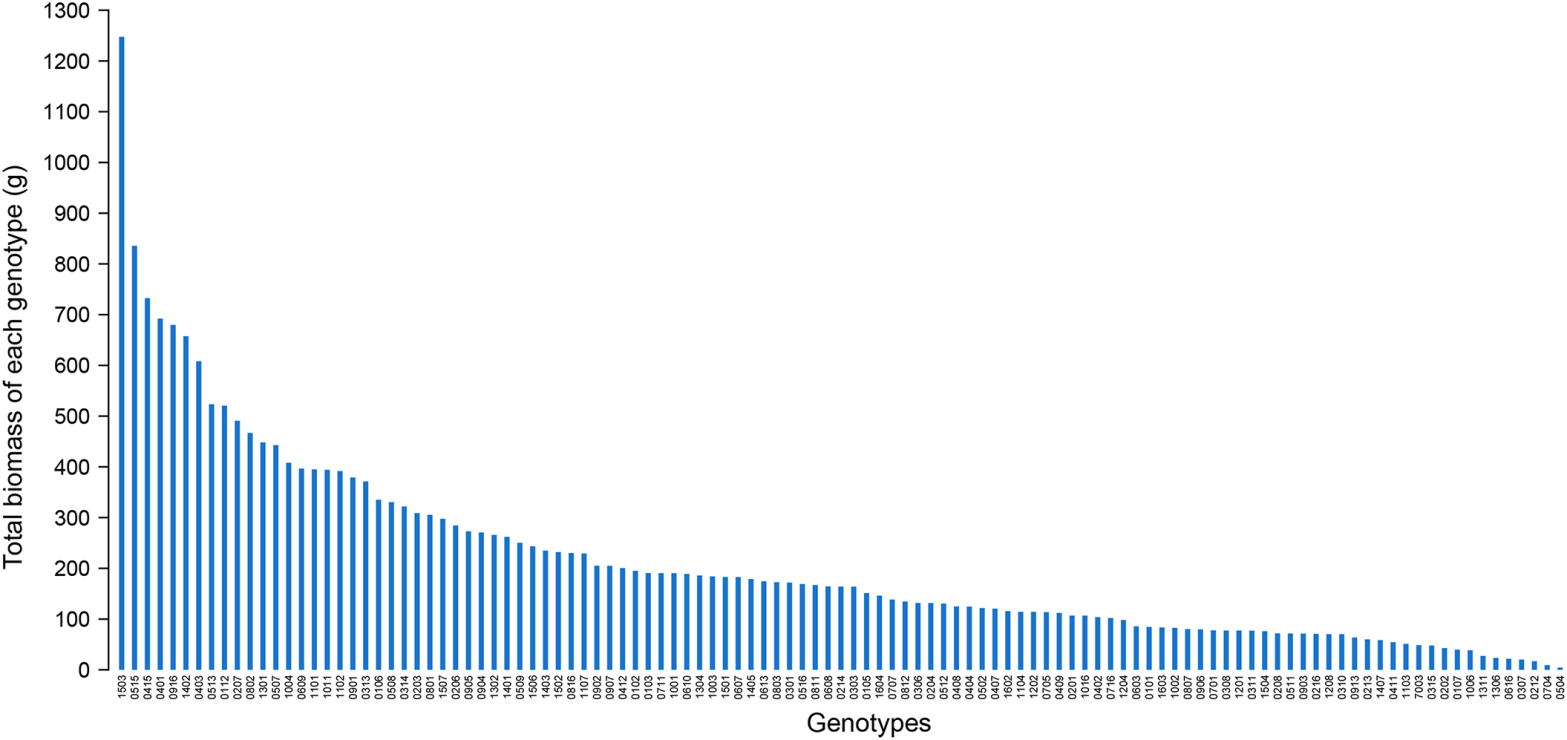
The distribution of the total biomass of all survival ones from 10 individuals planted in 10 m^2^ for each hybrid genotype in November 2019.

The total biomass of all surviving individuals for each hybrid genotype showed great variation and ranged from 4.59 g (genotype 0504) to 1247.49 g (genotype 1503) (**Figure 5**). The total biomass of genotype 1503 outperformed other hybrid genotypes. On one hand, it was 6 times heavier than the average total biomass of 213 g. On the other hand, it was 1.5 times heavier than that of the second genotype 0515 (835.74 g). Because the distribution of the total biomass was severely skewed, the first seven genotypes were prominent and can be selected as candidate genotypes with high biomass and establishment rate.

Meanwhile, it was worth noting that there was a large variation in morphological and physiological traits between hybrid genotypes at the end of the second growing season. The largest variation was found in the branch number of hybrid genotypes, which was followed by plant biomass, tiller number, biomass of individual tillers, and WUE_e_ (**Table 1**). A similar pattern was observed in the variation of hybrid genotypes at the end of the 2018 growing season (**Supplementary Table 1**). Therefore, these variations of multiple traits provided the base of selection for breed interspecific hybrid with stronger adaptability and higher biomass.

**Table 1:** The trait variation within hybrid genotypes at the end of the 2018 growing season.

Traits	Min	Max	Mean	*SDEV*	*CV*(%)
Plant height (cm)	98.67	200.67	147.18	21.38	14.52
Stem height (cm)	51.00	129.00	87.42	18.69	21.38
Stem diameter (mm)	3.80	9.73	6.77	1.20	17.80
Tiller number	1	13	4.13	2.57	62.21
Node number	9	16	13.05	1.71	13.09
Branch number	0	5	0.49	0.86	174.89
Internode length (cm)	4.33	10.50	7.67	1.21	15.83
Leaf width (cm)	0.97	1.90	1.36	0.20	14.81
Leaf area (cm^2^)	29.65	114.21	57.35	12.87	22.45
Leaf base angle (°)	9.00	29.00	17.92	4.47	24.92
Biomass of individual tillers (g)	2.38	28.24	10.63	4.99	46.92
Plant biomass (g)	3.39	138.61	31.19	25.00	80.16
*A* (μmolCO_2_m^-2^s^-1^)	9.09	34.18	19.57	3.69	18.84
*g_s_* (mmolCO_2_m^-2^s^-1^)	0.06	0.25	0.14	0.03	21.32
*C_i_* (μmol mol^-1^)	56.79	181.07	135.16	19.29	14.27
*E* (mmolH_2_Om^-2^s^-1^)	1.73	7.19	3.99	0.97	24.39
WUE_e_ (mmol mol^-1^)	3.16	10.39	5.07	1.16	22.86
WUE_i_ (μmol mol^-1^)	117.08	192.80	142.78	12.11	8.48

A total of 333 individuals of 113 hybrid genotypes are calculated. Min and Max represent the minimum and maximum in all values of traits measured, respectively. Mean is the average value to all values of traits measured. SDEV is the abbreviation of standard deviation. CV represents the variable coefficient. A: Photosynthetic rate, g_s_: Stomatal conductance, C_i_: Intercellular CO_2_ concentration, E: Transpiration rate, WUE_e_: extrinsic water use efficiency (A/E), WUE_i_: intrinsic water use efficiency (A/g_s_).

First, the membership function analysis suggested that the integrated score of genotype 1502 was the highest and genotype 0212 was the lowest in all hybrid genotypes using membership function evaluation method. If the first 5% of integrated score was used to be the criterion, genotype 1502, 0513, 1503, 0112, 0802, and 1003 were selected as candidate genotypes. If the first 10% was used, genotype 1502, 0513, 1503, 0112, 0802, 1003, 1107, 0515, 0907, 1402, and 0313 were selected (**Supplementary Figure 6A**).

Second, the principal component analysis showed that the eigenvalues of the first three principal components were successively 6.54, 1.36, and 1 and the contribution values were 54.51%, 11.34%, and 8.37%, respectively (**Supplementary Table 2**). The loading matrices, score coefficient matrix, and the integrated score of three principal components were shown in **Supplementary Tables 3–5**, respectively. The weights of the first three principal components were calculated to 73.45%, 15.28%, and 11.28%. Therefore, the integrated scores of the first three principal components in each genotype were calculated and exhibited in **Supplementary Figure 6B**. It showed that genotype 0513 had the highest integrated score (4.19) and genotype 0212 had the lowest integrated score (-3.58) in all 113 hybrid genotypes. When the first 5% was used to be the criterion, the selected candidate genotypes were exactly the same as the membership function analysis. When the first 10% was used, genotype 0513, 1502, 1503, 1003, 0112, 0802, 0515, 1402, 0313, 0907, and 0507 were selected, which were 81.82% overlap by the two analyses above.

**Figure 6:**
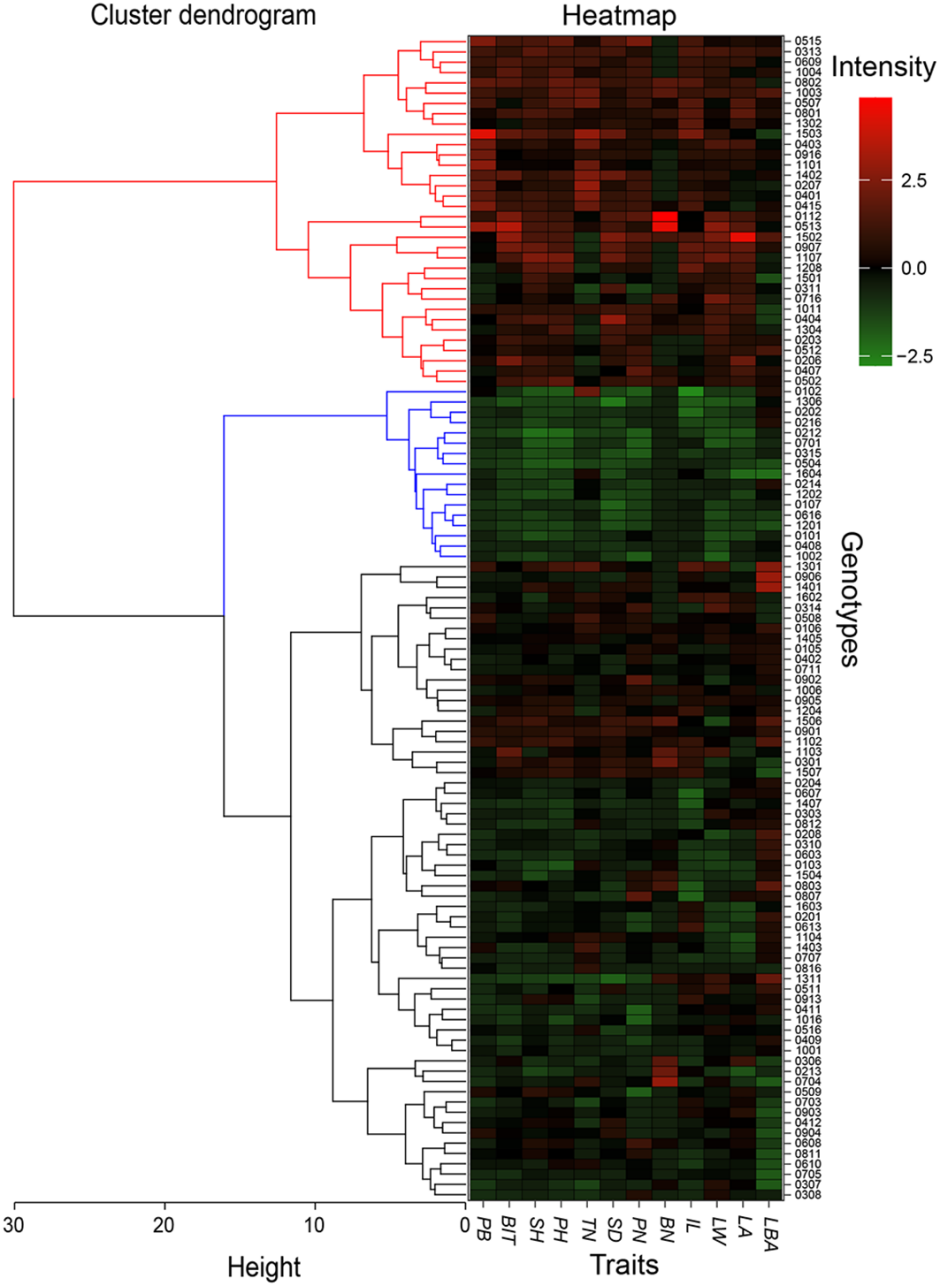
Systematic cluster of hybrid genotypes based on growth traits at the end of 2019 growing season. A total of 113 hybrid genotypes are divided into three classes that are showed by using the red, blue, and black lines on the left. The blocks from the red to the green in the heat map represent the good and poor growth performance, respectively. *PB*, plant biomass; *BIT*, biomass of individual tiller; *SH*, stem height; *PH*, plant height; *TN*, tiller number; *SD*, stem diameter; *NN*, Node number; *BN*, branch number; *IL*, internode length; *LW*, leaf width; *LA*, leaf area; *LBA*, leaf basic angle.

Finally, 113 hybrid genotypes were systematically clustered based on the plant biomass and biomass of individual tiller, and the other 10 growth traits measured at the end of the 2019 growing season. The 113 genotypes were grouped into 3 classes (**Figure 6**). A total of 34 hybrid genotypes performed well, which were divided into the first category genotype. The second class included 17 genotypes that showed poor growth traits with lower biomass. The rest was identified as the third class. Compared the first 34 and 17 hybrid genotypes with the candidates from other methods, respectively. The intersect included a total of 29 F_1_ hybrid genotypes (*M*_1_) with outstanding traits and 15 F_1_ genotypes (*M*_2_) with poor growth performance. *M*_1_ genotypes were identified as candidates for future energy crop development.

To study the difference of photosynthetic parameters between *M_1_* and *M_2_* genotypes, the One-way ANOVA was conducted. The results showed that photosynthetic rate and WUE_e_ of *M_1_* genotypes were significantly higher than that of *M_2_* genotypes in July in 2018 and 2019 when there was appeared drought and high temperature in the surface mining area (**Table 2**). It is worth noting that WUE_i_ of *M_1_* genotypes was higher than that of *M_2_* genotypes in each growth month when they were measured. It suggests that high WUE_i_ could contribute to the hybrids to grow well.

**Table 2:** One-way ANOVA on photosynthetic parameters of hybrid genotypes.

Traits	2018 growing season				2019 growing season			
	*M_1_*-*M_2_*	df	*F*	*P*-value	*M_1_*-*M_2_*	df	*F*	*P*-value
July								
*A* (μmol CO_2_ m^-2^ s^-1^)	+	1	4.847	0.030	+	1	10.310	0.002
*g_s_* (mmol CO_2_ m^-2^ s^-1^)	+	1	2.947	0.089	+	1	7.726	0.006
*C_i_* (μmol mol^-1^)	–	1	0.967	0.327	–	1	4.828	0.030
*E* (mmol H_2_O m^-2^ s^-1^)	+	1	0.274	0.601	+	1	0.021	0.885
WUE_e_ (mmol mol^-1^)	+	1	5.987	0.016	+	1	14.530	0.000
WUE_i_ (μmol mol^-1^)	+	1	0.581	0.447	+	1	0.731	0.394
August								
*A* (μmol CO_2_ m^-2^ s^-1^)	+	1	1.585	0.211	+	1	3.998	0.048
*g_s_* (mmol CO_2_ m^-2^ s^-1^)	+	1	0.434	0.512	+	1	2.640	0.107
*C_i_* (μmol mol^-1^)	–	1	3.667	0.058	–	1	6.379	0.013
*E* (mmol H_2_O m^-2^ s^-1^)	–	1	2.703	0.103	+	1	4.996	0.027
WUE_e_ (mmol mol^-1^)	+	1	6.866	0.010	–	1	0.729	0.395
WUE_i_ (μmol mol^-1^)	+	1	1.455	0.230	+	1	0.120	0.729
September								
*A* (μmol CO_2_ m^-2^ s^-1^)	+	1	1.673	0.198	–	1	0.029	0.865
*g_s_* (mmol CO_2_ m^-2^ s^-1^)	+	1	0.933	0.336	–	1	0.017	0.897
*C_i_* (μmol mol^-1^)	–	1	1.522	0.220	–	1	2.689	0.104
*E* (mmol H_2_O m^-2^ s^-1^)	+	1	0.885	0.349	–	1	0.067	0.796
WUE_e_ (mmol mol^-1^)	+	1	0.039	0.845	+	1	0.001	0.977
WUE_i_ (μmol mol^-1^)	+	1	0.488	0.486	+	1	0.044	0.834

M_1_ presents the value of the photosynthetic parameters measured in the genotypes with excellent comprehensive growth traits. M_2_ presents the value of the photosynthetic parameters measured in the genotypes with poor growth performance. + represents that the photosynthetic parameter of those hybrid genotypes with excellent comprehensive growth traits is more than those genotypes with poor growth performance. – represents that the photosynthetic parameter of those hybrid genotypes with excellent comprehensive growth traits is less than those genotypes with poor growth performance.

## Discussion

### Adaptation of hybrid genotypes to the stressful environment in the mine site

In this study, the high-biomass parent *M. lutarioriparius* failed to overwinter at -13.9°C monthly average minimum soil temperature in the field site while 113 *Miscanthus* hybrid genotypes were successfully established and maintained the survival rate 10%~100% and overwinter rate 66.67%~100%. As for morphological traits, plant height, stem height, tiller number, and stem diameter of hybrid genotypes were significantly better than those of the parents. Likewise, the photosynthetic rate of the hybrids was significantly higher than that of the parents, especially in the growth month with low temperature. Therefore, the hybrids of *M. sacchariflorus* and *M. lutarioriparius* showed stronger adaptability and greater production potential than their parents in harsh environments.

At the same time, the study revealed that hybrid genotypes grew faster in August with both sufficient precipitation and high temperature than in other months with drought or low temperature (**Figure 2**), which suggested that drought or low temperature could limit the growth of *Miscanthus* (Ings et al., 2013; Glowacka et al., 2014). Although there was a similar temperature in July and August during both 2018 and 2019, faster growth of plant height was observed in August rather than in July. It indicated that drought may limit the growth of *Miscanthus* in July. Similarly, there was a similar rainfall in August and September in 2019, but the growth of plant height was faster in August than in September. Together with the failed flowering of hybrid genotypes, the lower temperature in September could be the main reason explaining the slow vegetative growth in the experimental site. Harsh conditions may have a double negative effect on *Miscanthus* growth because the reduction in photosynthetic rate resulting from the decreased growth limited by drought or cold could contribute to the further decrease in plant growth (Ings et al., 2013; Glowacka et al., 2014).

Although this study over two years is relatively short for the field experiment or perennial grass, it was sufficient for the identification of the most suitable genotypes (Yan et al., 2012; Moon et al., 2018), partly because the first winter may be the most serious limiting factor for *Miscanthus* establishment from small rhizome cuttings (Clifton-Brown and Lewandowski, 2000). To thoroughly evaluate multiple yield-related traits, as many key morphological traits as possible were collected, including plant biomass, stem biomass, plant height, stem height, node number, tiller number, stem diameter, internode length, branch number, leaf area, leaf width, and leaf base angle. Also, the main physiological traits as much detail as possible were measured. More importantly, 1160 individuals were continuously monitored for each month during two growing seasons. Thus, two-year observations have made these results repeatable and credible to a large extent. As a preliminary work, this study has provided useful information on the cultivation and breeding of *Miscanthus* in the marginal land for bioproducts for sustainable bioeconomy. The ongoing experiment will provide more valuable information in the future.

### Selecting hybrid genotypes with key traits

Previous work has indicated that tiller diameter, plant height, and leaf width were correlative with biomass yield (Huang et al., 2011). Our study indicated that there was a significant correlation between plant biomass and plant height, tiller number, and leaf width (R^2^ = 0.76, *P*<0.001), respectively. Besides, the stem diameter, and branch number, and leaf width were individually significantly related to the biomass of individual tillers (R^2^ = 0.83, *P*<0.001). Hence, plant height, tiller number, stem diameter, branch number, and leaf width were identified as the key growth traits relative to the plant biomass.

Our study found that the leaf width of *Miscanthus* was correlative positively to plant biomass (r=0.73, *P*<0.001). Also, the leaf width of *Miscanthus* was positively related to the plant height, stem height, tiller number, and stem diameter, respectively (**Supplementary Figure 4**). Therefore, the average leaf width was a significant phenotype trait for selecting *Miscanthus* genotypes. In this study, the leaf width of hybrid genotypes was significantly larger than that of surviving parent *M. sacchariflorus*, which showed the advantage in the production potential of hybrid genotypes.

It was worth noting that a significant positive relationship between stomatal conductance and photosynthetic rate of *Miscanthus* hybrids has been shown in the study (*P*<0.001, **Supplementary Figure 4**). Stomatal closure due to drought stress could lead to a decrease in photosynthetic rate, intensifying carbon starvation, and even plant mortality (McDowell et al., 2008). Also, high stomatal conductance contributed to increasing photosynthetic rate and biomass yield (Pires et al., 2020). Hence, stomatal conductance is a physiological indicator that mirrored not only the stomatal conductance but also the photosynthetic rate. Although the high photosynthetic rate is due to the high stomatal conductance, it can increase the expense of additional transpiration (Dohleman et al., 2009). It was acknowledged that small stomatal conductance was more conducive to coping with the poor external environment (Leakey, 2009; Zhao et al., 2021). The plant could have suffered severe drought stress when the stomatal conductance decreased (Ings et al., 2013). Genotypes with lower stomatal conductance may be more resistant to drought stress but it may be hard to improve the biomass yield. In the study, *M*_1_ genotypes showed a significantly higher stomatal conductance in drought July in 2019 than *M*_2_ genotypes with poor growth performance (*P*<0.01, **Table 2**). This indicated that maintaining a high stomatal conductance under drought conditions could promote partially the growth and improve the biomass yield. It implied that *M*_1_ genotypes could be identified as the insensitive genotypes to drought stress while *M*_2_ were the sensitive genotypes.

### Interspecific hybridization as an effective approach for high-yield *Miscanthus* on the marginal land

*Miscanthus* has been expected to have strong adaptability to the arid and cold climates as well as barren soil (Yan et al., 2012; Fan et al., 2015; Fonteyne et al., 2016). The survival rate and overwinter rate were the important indicators in the adaptation process of *Miscanthus* populations to new habitats. If these two indicators of original breeds were too low, the interspecific hybridization would be an ideal direction to try to make adaptive genetic improvement (Dong et al., 2019). Recently, researchers have tried to overcome the adverse effects of low temperature by interspecific hybrids of *Miscanthus* (Glowacka et al., 2014; Fonteyne et al., 2016; Moon et al., 2018). Due to the increased overwinter rate in our study, interspecific hybrids were expected to play an important role in the practical production of *Miscanthus*. It could be that parental genotypes endowed hybrids with the complementary character of higher biomass and stronger cold tolerance (Tamura et al., 2016; Lee et al., 2022) and that thus could be more favorable for the ecological restoration on the reclaimed mine land. Therefore, it implied that new genotypes with stronger adaptability can be cultivated through interspecific hybridization of *Miscanthus*.

Although the variation of establishment rate between F_1_ genotypes posed a great effect on the selection, harvesting high biomass yields is the main goal for the raw material production of biofuels (Robson et al., 2013b). On one hand, the parent *M. lutarioriparius* with high biomass yield was selected. On the other hand, the parent *M. sacchariflorus* with high tolerance was selected because harsh conditions result in the great loss of plant biomass. A large variation in plant biomass (80.16%) and biomass of individual tillers (46.92%) between hybrid genotypes may make it easier for selecting the genotypes with high biomass and strong tolerance to multi-stressful environments. These significant differences in growth traits between genotypes suggested the abundant genetic diversity of hybrids and were consistent with the principal component analysis (**Supplementary Figure 5**).

It has been reported that the biomass of *Miscanthus* increased after planting in the first three years (Heaton et al., 2008), which was consistent with our results (R^2^ = 0.47, *P*<0.01). Meanwhile, the plant biomass and biomass of individual tillers were significantly larger in 2019 than in 2018 (*P*<0.05), as was shown in the previous studies (Robson et al., 2013a). Due to the instability of growth during the establishment period, the increase in leaf area in harsh conditions may contribute partially to a higher biomass of *Miscanthus* in 2019 (**Supplementary Figure 1C**). Moreover, *Fv’/Fm’*, *ΦPSII*, *qP*, and *NPQ* of the leaf except for *φCO_2_* and *ETR* were significantly higher in 2019 than in 2018 (**Supplementary Figure 7**), which indicated a stronger photosynthetic ability in the second growing season. These increases in fluorescence parameters could relate to the robust growth of hybrid genotypes in 2019.

Finally, the index “Total biomass of each genotype (g)” in **Figure 5** was not the yield estimate of the hybrids, but the score was used to identify the genotypes with outstanding traits as candidates for energy crop development. In fact, the data that we obtained in 2019 was from a field experiment with a plant density of 1 m^-2^ per individual. However, the practical field trials were designed in the agricultural setting with a much higher plant and tiller density. Based on our observation, the tiller density fell predominantly from 40 m^-2^ in the natural habitat to 20 m^-2^ in the planted field (Yan et al., 2012; Zheng et al., 2019). If we used the mean tiller density of 30 m^-2^ for calculating the potential yield of the hybrids, the highest yield is 37.4 t/ha (genotype 1503) and the mean yield is 6.4 t/ha. Considering that the experiment was still preliminary plot trials, the interspecific hybrids of *Miscanthus* showed great production potential in harsh environments.

## Conclusion

In this study, a total of 29 interspecific F_1_ hybrids with outstanding traits related to yield and stress tolerance were identified as candidates for future energy crop development on the mine land of the Loess Plateau. These hybrid genotypes clearly outperformed their parents for establishment rates, biomass yield, photosynthetic rates, and water use efficiency. The study demonstrated that hybridization between an individual of *M. sacchariflorus* adapting to the local climates and high-biomass *M. lutarioriparius* could provide an effective approach for developing high-yield energy crop on marginal lands with harsh climate and soil conditions.

## Data availability statement

The raw data supporting the conclusions of this article will be made available by the authors, without undue reservation.

## Author contributions

WL contributed to the conception and design of the study. LX, HH, and ZY provided the plant materials. XZ, LK, CL, JM, and JY contributed to complete the experiment. XZ and WL performed the statistical analysis. XZ and LX wrote the first draft of the manuscript. WL, JM, TS and WC revised the manuscript. TS and ZY made substantial guide about experiment design, and critically revised the manuscript. All authors read and approved the final manuscript.
