# Supplementary material for: Development of energy plants from hybrids between Miscanthus sacchariflorus and M. lutarioriparius grown on reclaimed mine land in the Loess Plateau of China
Source: Front Plant Sci. 2023 Jan 16;13:1017712. doi: 10.3389/fpls.2022.1017712 (PMC9885154; doi:10.3389/fpls.2022.1017712)
Supplement: Supplementary file 1 [file DataSheet_1.pdf]

Supplementary Material

Supplementary Figures and Tables

Supplementary Figures

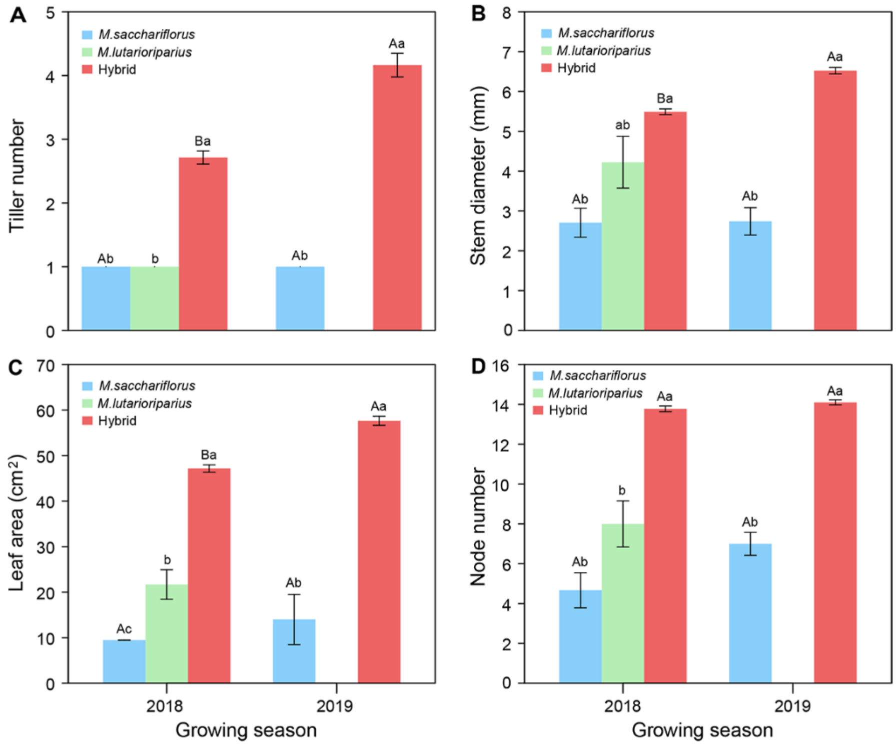

Supplementary Figure 1. Differences of (A) average tiller number, (B) stem diameter, (C) leaf area and (D) node number between measuring hybrid and parent individuals in two consecutive growth seasons. Different capital letters represent a significant difference between two years at *P*<0.05 level, and different lowercases represent a significant difference between three *Miscanthus* at *P*<0.05 level. Error bars indicate standard error of 333 surviving hybrid individuals, 5 and 3 surviving female parent individuals in 2018 and 2019, and 10 surviving male parent individuals in 2018, respectively.

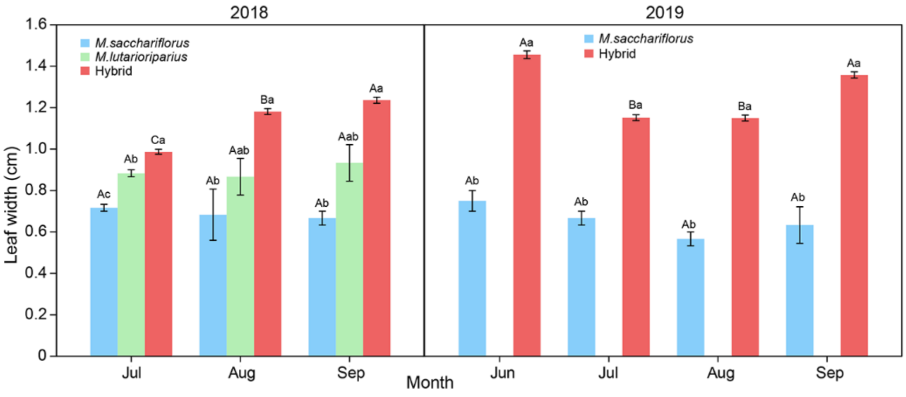

Supplementary Figure 2. Differences of average leaf width between measuring hybrid and parent individuals throughout growth months in two growing seasons. Different capital letters represent a significant difference in leaf width between growth months at $P<0.05$ level and different lowercases represent a significant difference of three *Miscanthus* at $P<0.05$ level. Error bars indicate standard error of 333 surviving hybrid individuals, 5 and 3 surviving female parent individuals in 2018 and 2019, and 10 surviving male parent individuals in 2018, respectively.

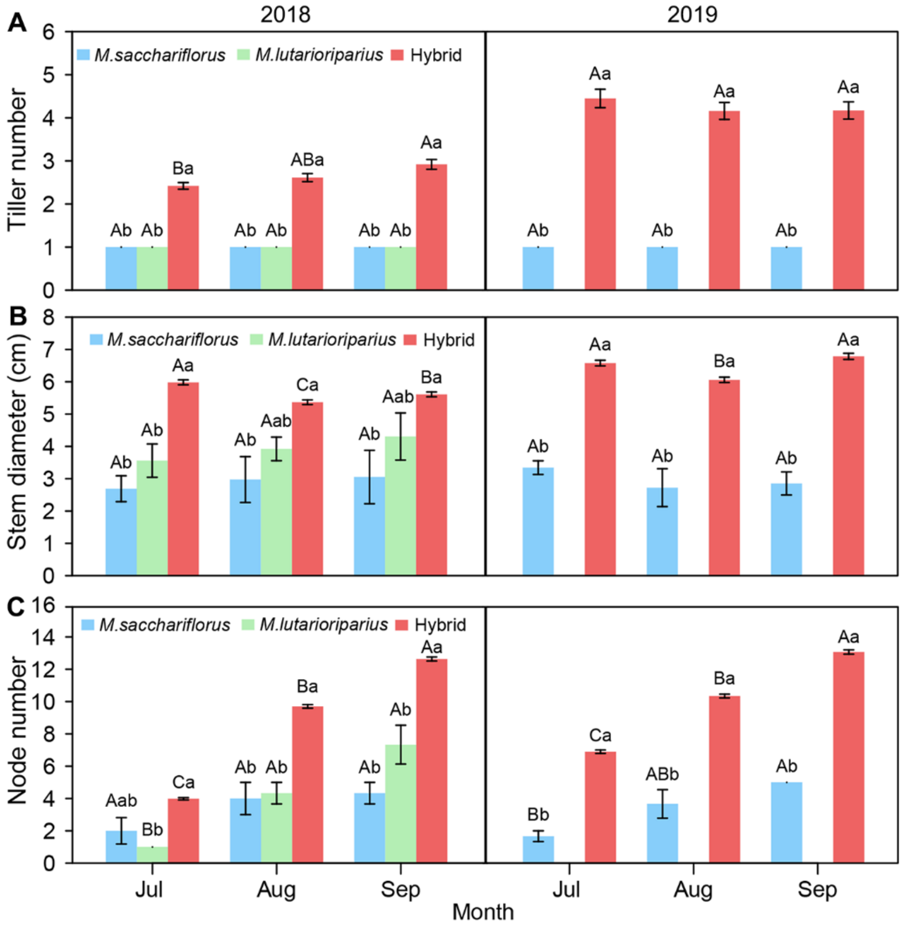

Supplementary Figure 3. Differences of (A) average tiller number, (B) stem diameter and (C) node number between measuring hybrid and parent individuals in the 2018 and 2019 growing season. Different capital letters represent a significant difference between growth months at *P*<0.05 level, and different lowercases represent a significant difference in tiller number, stem diameter and pitch number between three *Miscanthus* at *P*<0.05 level. Error bars indicate standard error of 333 surviving hybrid individuals, 5 and 3 surviving female parent individuals in 2018 and 2019, and 10 surviving male parent individuals in 2018, respectively.

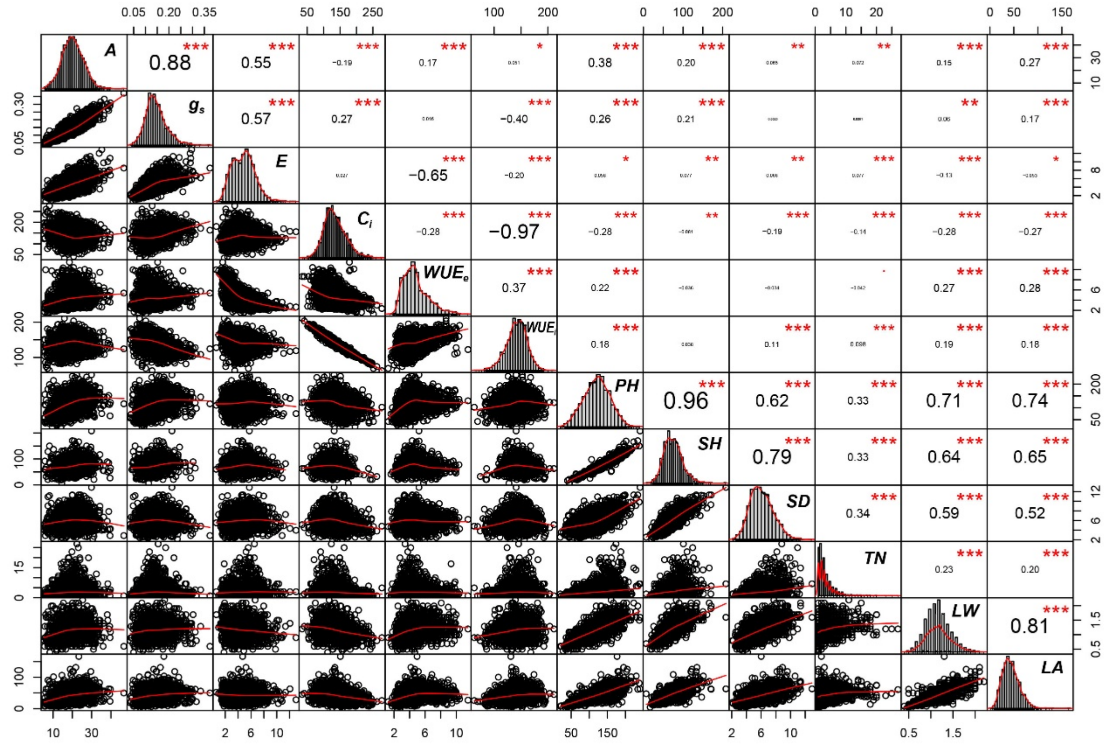

Supplementary Figure 4. Correlation analysis between the growth traits and photosynthetic parameters. The histograms in diagonal line show the whole distribution character in growth and photosynthetic parameters of hybrid genotypes during the 2018 and 2019 growing season. The red asterisks show the significant correlation between each two traits. The figures are the coefficient of association between each two traits. The scatter diagrams in bottom left show the pictorial diagram of each two traits. *A*: phorosynthetic rate. g_s: stomatal conductance. *E*: transpiration rate. C_i: intercellular CO_2 concentration. WUE_e: extrinsic water use efficiency. WUE_i: intrinsic water use efficiency. *PH*: plant height. *SH*: stem height. *SD*: stem diameter. *TN*: tiller number. *LW*: leaf width. *LA*: eaf area. * represents the correlation at *P*<0.05 level. ** represents the correlation at *P*<0.01 level. *** represents the correlation at *P*<0.001 level.

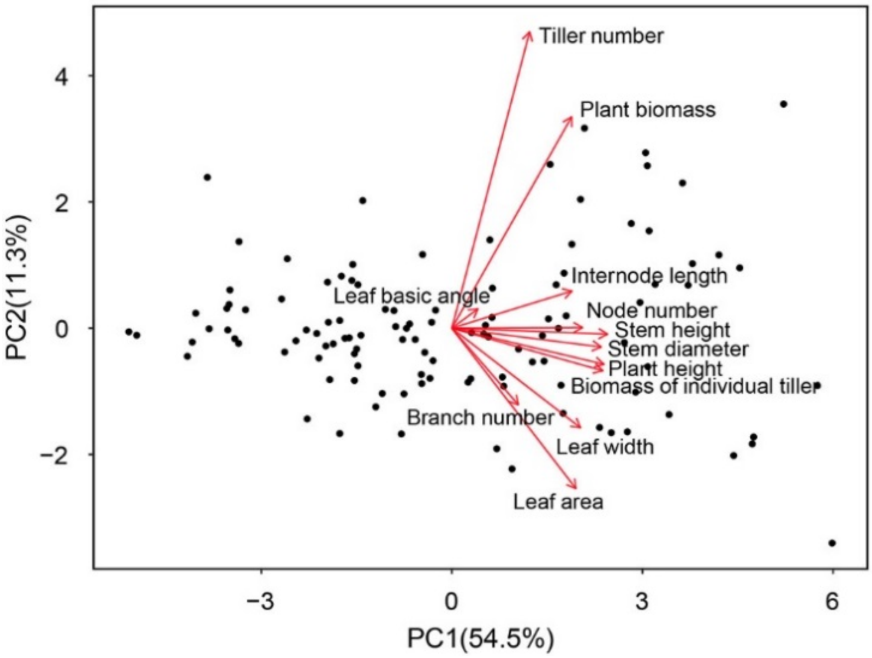

Supplementary Figure 5. The principal component analysis in growth traits of hybrid genotype. The black dots represent the hybrid genotypes in the study. The red arrows show the response of growth traits to the principal component.

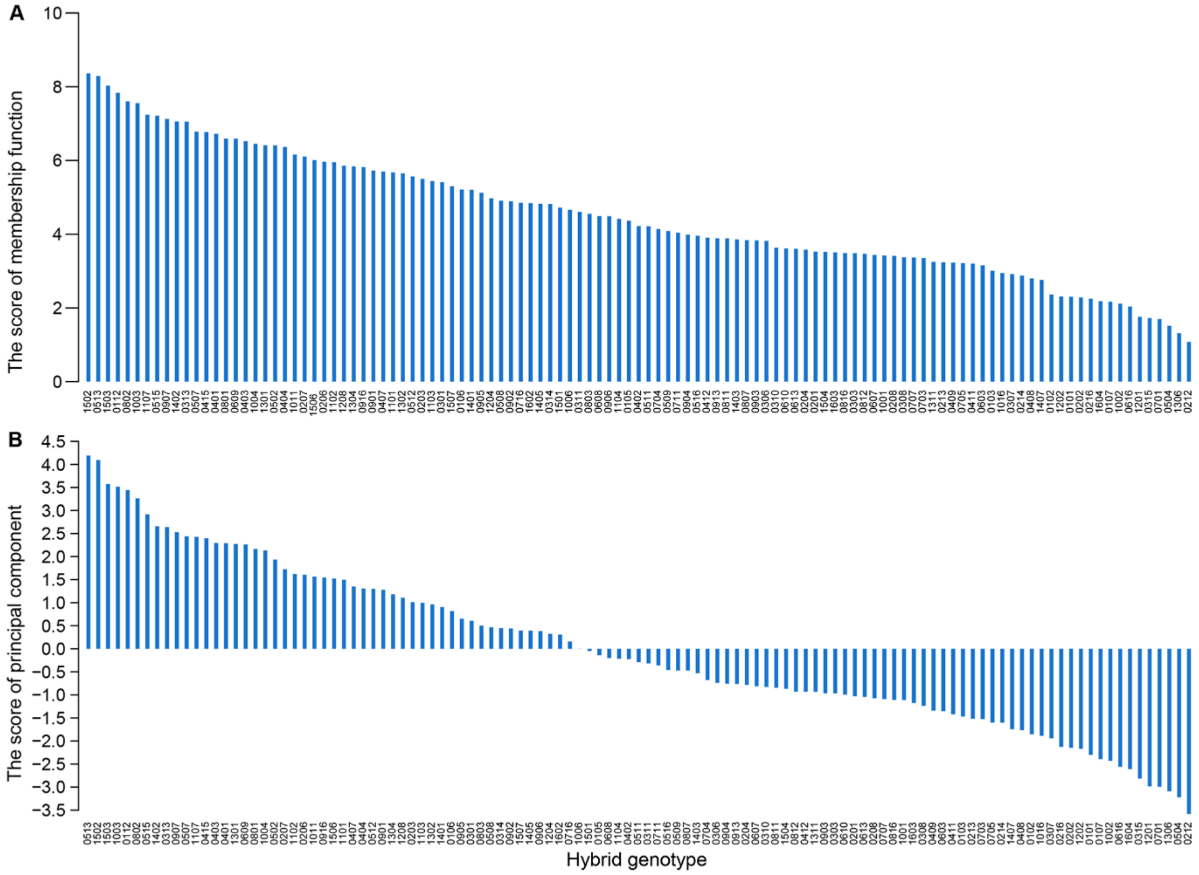

Supplementary Figure 6. The integrated score using (A) the membership function, (B) principal component for each hybrid genotype.

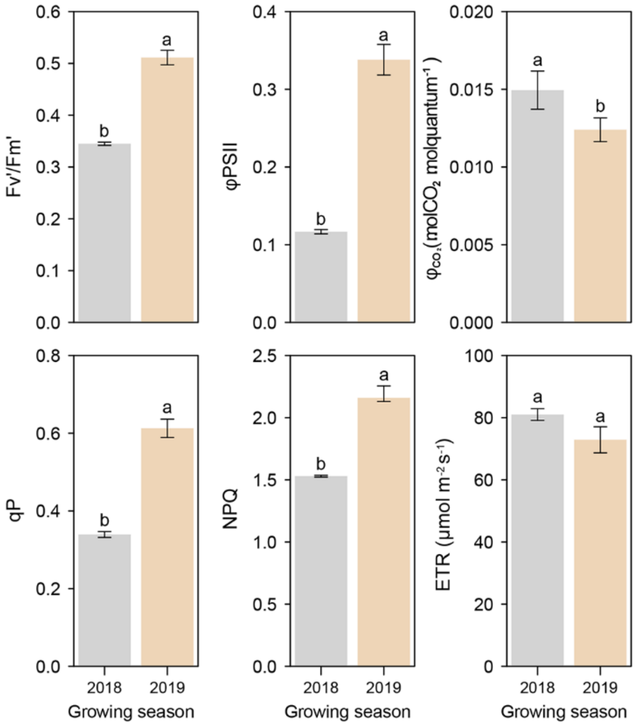

Supplementary Figure 7. The fluorescence parameters of hybrid leaf in the most vigorous growth months. The different lowercases represent a significant difference between the growing seasons at *P*<0.05 level. Error bars indicate standard error of 333 surviving hybrid individuals in 2018 and 2019, respectively.

Supplementary tables

Supplementary Table 1. The trait variation within hybrid genotypes at the end of the 2018 growing season. A total of 333 individuals of 113 hybrid genotypes are calculated. Min and Max represent the minimum and maximum value in all traits measured, respectively. Mean is the average value to all values of traits measured. *SDEV* is the abbreviation of standard deviation. *CV* represents the variable coefficient. A: Photosynthetic rate. g_s: Stomatal conductance. C_i: Intercellular CO_2 concentration. E: Transpiration rate. WUE_e: extrinsic water use efficiency (A/E). WUE_i: intrinsic water use efficiency (A/g_s).

Traits	Min	Max	Mean	*SDEV*	*CV*(%)
Plant height (cm)	73.33	153.00	116.55	17.92	15.38
Stem height (cm)	26.54	92.36	62.39	13.95	22.36
Stem diameter (mm)	2.60	7.89	5.59	1.04	18.61
Tiller number	1	10	2.93	1.58	54.10
Node number	8	16	12.62	1.80	14.23
Branch number	0	8	1.68	1.51	90.17
Internode length (cm)	3.75	11.00	6.98	1.32	18.91
Leaf width (cm)	0.73	1.63	1.23	0.19	15.74
Leaf area (cm^2)	22.63	73.51	47.12	10.91	23.16
Leaf base angle (°)	11.33	35.33	23.31	4.65	19.93
Biomass of individual tiller (g)	2.08	25.28	7.88	4.78	60.62
Plant biomass (g)	2.50	55.45	15.59	11.53	73.95
A ($\mu molCO_2m^{-2}s^{-1}$)	10.09	25.83	18.24	3.06	16.79
g_s ($mmolCO_2m^{-2}s^{-1}$)	0.06	0.17	0.12	0.02	18.12
C_i ($\mu mol\ mol^{-1}$)	49.30	212.91	116.98	27.47	23.49
E ($mmolH_2Om^{-2}s^{-1}$)	0.88	4.32	2.77	0.66	23.92
WUE(A/E) ($mmol\ mol^{-1}$)	4.99	11.27	6.82	1.28	18.77
WUE(A/g_s) ($\mu mol\ mol^{-1}$)	101.59	201.33	157.68	16.03	10.17

Supplementary Table 2. Total variance in the interpretation.

Principal component	Initial Eigenvalues			Extraction Sums of Squared Loadings		
	Total	Percentage of variance	Percentage of accumulation	Total	Percentage of variance	Percentage of accumulation
1	6.54	54.51	54.51	6.54	54.51	54.51
2	1.36	11.34	65.85	1.36	11.34	65.85
3	1.00	8.37	74.22	1.00	8.37	74.22
4	0.97	8.09	82.31			
5	0.58	4.79	87.10			
6	0.53	4.37	91.48			
7	0.28	2.32	93.79			
8	0.26	2.15	95.94			
9	0.17	1.39	97.33			
10	0.16	1.35	98.68			
11	0.10	0.86	99.54			
12	0.06	0.46	100.00			

Supplementary Table 3. Loading matrix of principal component.

Traits	Comp.1	Comp.2	Comp.3
Biomass of individual tiller (g)	0.907	-0.117	-0.065
Plant biomass (g)	0.720	0.582	-0.087
Tiller number	0.467	0.817	-0.094
Stem diameter (cm)	0.897	-0.052	-0.050
Leaf width (cm)	0.772	-0.274	-0.068
Node number	0.785	0.002	0.114
Leaf area (cm^2)	0.745	-0.442	0.002
Branch number (cm)	0.399	-0.211	-0.272
Internode length (cm)	0.721	0.102	0.018
Leaf basic angle (°)	0.156	0.053	0.937
Plant height (cm)	0.913	-0.099	0.098
Stem height (cm)	0.935	-0.016	0.032

Supplementary Table 4. The score coefficient matrix of principal component.

Traits	Comp.1	Comp.2	Comp.3
Biomass of individual tiller (g)	0.354	-0.100	-0.065
Plant biomass (g)	0.281	0.499	-0.087
Tiller number	0.183	0.700	-0.093
Stem diameter (cm)	0.351	-0.040	-0.050
Leaf width (cm)	0.302	-0.235	-0.068
Node number	0.307	0.002	0.114
Leaf area (cm^2)	0.291	-0.379	0.002
Branch number (cm)	0.156	-0.181	-0.272
Internode length (cm)	0.282	0.088	0.018
Leaf basic angle (°)	0.061	0.046	0.935
Plant height (cm)	0.357	-0.085	0.098
Stem height (cm)	0.365	-0.014	0.032

Supplementary Table 5. The integrate score of each principal component.

Genotypes	Comp.1	Comp.2	Comp.3	Genotypes	Comp.1	Comp.2	Comp.3
0513	6.107	-0.572	-1.835	0314	0.745	-0.166	-0.658
1502	6.123	-3.470	1.131	0902	0.450	0.151	0.769
1503	4.437	3.530	-1.986	1507	0.752	0.131	-1.569
1003	4.451	0.995	0.830	1405	0.523	0.002	0.089
0112	5.161	-1.632	-0.891	0906	0.033	-0.020	3.212
0802	4.427	0.925	-1.146	1204	0.539	-0.937	0.624
0515	3.658	1.133	0.511	1602	0.496	0.021	-0.534
1402	3.135	2.285	0.050	0716	0.736	-1.837	-0.911
0313	3.325	0.648	0.900	1006	0.216	-0.826	-0.265
0907	3.752	-2.058	0.792	1501	0.273	-0.804	-1.122
0507	2.973	1.503	0.233	0105	-0.169	-0.541	0.605
1107	3.699	-1.761	-0.162	0608	0.042	-0.877	-0.861
0415	2.688	2.769	0.010	1104	-0.603	1.167	0.423
0403	2.851	1.623	-0.425	0402	-0.386	-0.204	0.807
0401	2.618	2.556	-0.200	0511	-0.357	-0.894	0.956
1301	2.303	2.031	2.408	0311	-0.041	-1.935	0.084
0609	2.946	0.662	-0.024	0711	-0.589	0.009	0.623
0801	3.032	-0.647	0.324	0516	-0.677	0.347	-0.129
1004	2.728	0.392	0.620	0509	-0.587	0.070	-0.448
0502	2.823	-1.043	0.189	0807	-0.564	-1.058	0.927
0207	1.789	3.153	-0.615	1403	-1.190	2.013	0.335
1102	1.836	0.861	1.265	0704	-0.494	-0.190	-2.524
0206	2.514	-1.684	0.167	0306	-0.399	-1.707	-1.629
1011	2.394	-0.263	-1.342	0904	-0.865	0.266	-1.452
0916	1.802	1.300	0.217	0913	-0.931	-1.054	0.719
1506	1.859	0.175	1.165	0204	-1.145	-0.128	0.690
1101	1.568	2.569	-0.410	0607	-1.128	-0.185	0.402
0407	2.031	-1.381	0.624	0310	-1.153	-0.606	0.974
0404	2.236	-1.397	-1.072	0811	-0.888	-0.403	-1.158
0512	1.662	-0.550	1.449	1504	-1.243	0.086	0.282
0901	1.516	0.668	0.558	0812	-1.367	0.097	0.502
1304	2.023	-1.600	-0.507	0412	-0.963	-0.816	-0.905
1208	1.830	-1.688	0.206	1311	-1.297	-1.068	1.596
0203	1.476	-0.927	0.621	0903	-0.961	-0.759	-1.284
1103	1.601	-0.547	-0.833	0303	-1.340	-0.131	0.312
1302	1.296	-0.034	0.155	0610	-1.204	0.283	-1.350
1401	0.764	-0.105	3.189	0201	-1.731	0.822	1.052
0106	0.870	0.617	0.762	0613	-1.669	0.675	0.690
0905	0.879	-0.352	0.537	0208	-1.640	-0.257	1.513
0301	1.097	-0.134	-1.576	0707	-1.706	0.721	0.463
0803	0.495	-0.092	1.373	0816	-1.618	0.995	-0.654
0508	0.453	1.379	-0.683	1001	-1.525	-0.166	0.298
1603	-1.782	0.753	0.165	0202	-2.949	-0.250	0.489
0308	-1.490	-0.795	-0.166	1202	-3.071	0.603	-0.045
0409	-1.862	-0.301	0.645	0101	-3.160	0.290	-0.213
0603	-1.996	-0.043	1.067	0107	-3.203	-0.173	-0.158
0411	-1.851	-0.831	0.593	1002	-3.359	0.358	-0.177
0103	-2.239	1.091	0.063	0616	-3.414	0.302	-0.877
0213	-1.849	-0.082	-1.292	1604	-3.547	1.373	-1.912

Continued Supplementary Table 5.

Genotypes	Comp.1	Comp.2	Comp.3	Genotypes	Comp.1	Comp.2	Comp.3
0703	-1.694	-1.265	-0.760	0315	-3.725	-0.229	-0.405
0705	-1.880	-0.419	-1.372	1201	-3.868	-0.015	-1.237
0214	-2.356	0.448	0.522	0701	-4.032	0.237	-0.630
1407	-2.297	-0.393	0.032	1306	-4.177	-0.335	0.260
0408	-2.394	-0.216	0.223	0504	-4.095	-0.452	-1.290
0102	-3.044	2.381	0.155	0212	-4.794	-0.119	-0.385
1016	-2.397	-0.499	-0.453				
